# Site-Specific Assembly of DNA-Based Photonic Wires by Using Programmable Polyamides**

**DOI:** 10.1002/anie.201006735

**Published:** 2011-02-25

**Authors:** Wu Su, Markus Schuster, Clive R Bagshaw, Ulrich Rant, Glenn A Burley

**Affiliations:** Department of Chemistry, University of LeicesterUniversity Road, Leicester, LE1 7RH (UK), Fax: (+44) 116-252-3789; Walter Schottky Institute, Technical University of MunichAm Coulombwall 3, 85748 Garching (Germany); Department of BiochemistryUniversity of Leicester Lancaster Road, Leicester LE1 9HN (UK)

**Keywords:** DNA, energy transfer, minor groove, photonic wire, polyamides

The bottom-up organization of functional materials with nanoscale precision is a central goal for the development of future sensors, machines, and devices.[1] Underpinning these developments has been the rapid progression of DNA-guided processes,[2] which uniquely provides an addressable template for the placement of molecular components in discrete one-, two-, and three-dimensional assemblies.[1d, 3] However, the incorporation of multiple molecular components such as fluorophores into such arrays reproducibly and with well-controlled molecular distances remains a formidable challenge.[4] Previous studies have highlighted the utility of DNA-based photonic wire systems,[5] however, these systems suffer from energy transfer (ET) losses associated with inefficient self-assembly of the appropriate communicating components along or within the DNA duplexes. In order to overcome these inefficiencies and provide the means to construct modular photonic wires of increasing complexity and addressability, we require tools which enable the exquisite control of the location and spatial arrangement of fluorophores within a DNA duplex.

Pyrrole-imidazole polyamides (PAs) are a class of small-molecule ligands which provide such control. PAs bind within the minor groove of duplex DNA, enabling one to target 6 to 10 base pair sequences with high binding affinity (nanomolar to subnanomolar) and specificity.[6] We surmised that interfacing the highly specific recognition properties of PAs with fluorophore relays, it would be possible to construct an addressable photonic wire model system where for the first time one could control the precise intercalation of a fluorophore within a DNA duplex with base pair level (i.e. 0.34 nm) precision.

**Figure 1:**
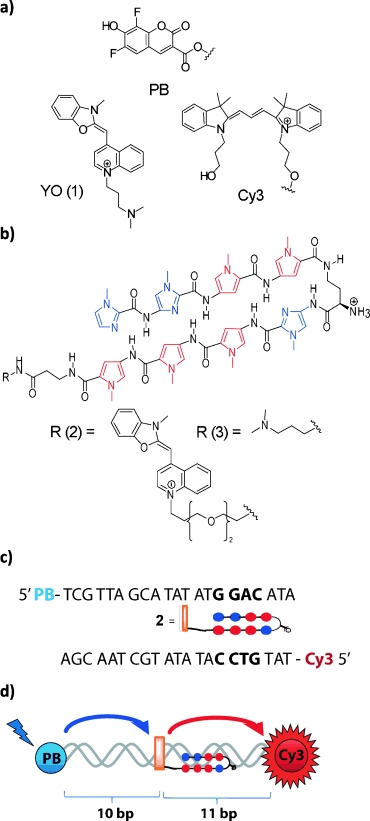
a) Chemical structures of the fluorophores PB (injector), Cy3 (reporter), and YO (**1**). b) Chemical structures of PAs **2** and **3**. c) Structural representation of the exemplar DNA-based photonic wire **2**@DNA21. The structure of PA **2** is represented as filled blue circles for imidazole (Im) building blocks whereas filled red circles represent pyrrole (Py) building blocks. d) Schematic representation of **2**@DNA21. The interfluorophore PB–YO distance is approximately 10 base pairs (ca. 3.4 nm) whereas the interfluorophore YO–Cy3 is approximately 11 base pairs (ca. 3.7 nm).

Here, we demonstrate this proof of concept with the programmable control of intercalating fluorophores within a duplex. The resultant PA-programmed assemblies exhibited enhanced and facile energy transport over distances in excess of 27 nm. We constructed an architecture comprising three fluorophores: Pacific Blue (PB), an intercalating cyanine dye (oxazole yellow; YO, **1**) and Cyanine 3 (Cy3, Figure 1a). The PB was used as the initial donor chromophore, whereas the Cy3 functions as a terminal energy acceptor.[5d] The ability of PAs to augment ET could then be tested through the mediation of the Förster resonance ET (FRET) response using a PA-tethered YO (**2**, Figure 1b,c) targeted to the specific binding site 5′-WWGGWCW-3′ (W=A or T) relative to **1**, which lacks sequence selectivity.[7] Three exemplar wire architectures were used in this study, differing in both the overall length and number of PA binding sites: DNA21 contains a single PA binding site (Figure 1c,d) whereas DNA55 and DNA80 comprise four and six PA binding sites, respectively (Supporting Information, Table S1 and Figure S2).[9]

An asymmetric-core PA sequence **3** was chosen, which exhibited both high binding affinity as well as sequence directionality for the specific binding site 5′-ATGGACA-3′.[7–8] PAs **2** and **3** were then prepared through a combination of standard solid phase synthesis protocols and a new triphosgene-activation approach (Figure S1).[9] The tether length utilized in the design of PA **2** separated the YO intercalator and the PA by two base pairs relative to the β-alanine tail terminus,[8b, 10] equating to an overall 9 base pair binding profile for PA **2**. We initially investigated the capacity of PA **2** to selectively bind to the sequence 5′-ATGGACA-3′ using isothermal binding and fluorescence enhancement measurements.[9] Isothermal binding studies revealed a high binding affinity of PA **2** for its target binding sequence as highlighted by a 16 °C duplex stabilization (Figure S3).[9] The sequence selectivity of PA **2** for this binding site relative to a sequence comprising a one base pair mismatch (5′-ATG*C*ACA-3′) was then investigated using a fluorescence enhancement assay.[9] YO (**1**) and PA **2** are virtually nonfluorescent in free solution equating to minimal contribution of unbound **1** and **2** to background fluorescence (Figure S4).[8] An intense YO emission at 509 nm was observed upon addition of PA **2** to a DNA duplex comprising the match sequence, whereas a 79.3 % drop in YO emission was observed in the presence of a DNA duplex containing the one base pair mismatch.[9] This is suggestive of the high sequence selectivity for PA **2** for its target binding sequence.

**Figure 2:**
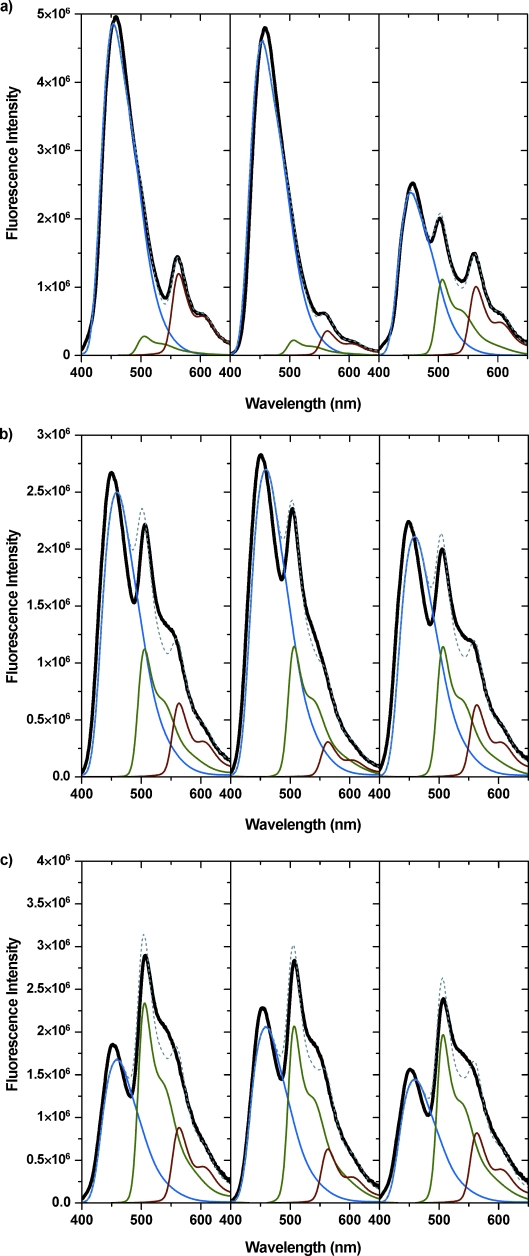
Steady-state spectra of a) **2**@DNA21, 1.0 equiv (left); **1**@DNA21, 1.0 equiv (center); **1**@DNA21, 3.0 equiv (right); b) **2**@DNA55, 4.0 equiv (left); **1**@DNA55, 4.0 equiv (center); **1**@DNA55, 12.0 equiv (right); c) **2**@DNA80, 6.0 equiv (left); **1**@DNA80, 6.0 equiv (center); **1**@DNA80, 18.0 equiv (right). Black line: steady-state emission spectra; blue line: deconvoluted PB emission; green line: deconvoluted YO emission; red line: deconvoluted Cy3 emission; dashed gray line: fitted emission spectra. All measurements were performed at 50 nm DNA.

The potential of PAs to construct addressable DNA-based photonic wire assemblies was then investigated using steady-state fluorescence emission measurements. Initial steady-state measurements of the control DNA21 duplex (i.e. in the absence of YO intercalator) revealed a dominant PB emission after PB excitation at 380 nm with very little Cy3 emission (570 nm) observed (Figure S5).[9] Upon addition of 1.0 equivalent of PA **2** (**2**@DNA21, where @ denotes the addition of **2** to DNA21) a threefold enhancement of the Cy3 emission (Figure 2a, left) was observed relative to an assembly comprising one equivalent of YO (**1**@DNA21, 1.0 equiv; Figure 2a, center). Deconvolution of the emission spectra of both assemblies revealed comparative levels of PB and YO emision, therefore we surmise that the enhancement of Cy3 emission observed for **2**@DNA21 is attributed to PA **2** augmenting efficient energy transport at the second energy transport step (i.e. from YO to Cy3) rather than exhibiting a major effect on the first energy transport step (i.e. from PB to YO).

Increasing the number of equivalents of YO (**1**) from one to three (**1**@DNA21, 3.0 equiv; Figure 2a, right) resulted in a decrease in PB emission and an increase in Cy3 emission to levels approaching that of the PA-containing assembly **2**@DNA21. A key difference however is the fourfold increase in YO emission for **1**@DNA21 (3.0 equiv). This is indicative of the extra energy injected into **1**@DNA21 (3.0 equiv) is being trapped within the non-sequence selective YO (**1**) dyes as a consequence of a heterogeneous ensemble of energy transfer events involving both hetero-FRET (i.e. ET from PB to YO and from YO to Cy3) and homo-FRET (i.e. ET between YO dyes) processes. In contrast, **2**@DNA21 is a discrete assembly in which the position of a single intercalating YO dye is controlled by PA binding to its target sequence and as a result, only a two-step hetero-FRET process (that is, PB→YO→Cy3) is observed. We therefore conclude that the PA-programming approach of directing the location of a single intercalating YO dye by PA binding to its orthogonal binding sequence in DNA21 increases the efficiency of energy transport through the DNA duplex.

Quantification of the end-to-end ET efficiencies of the **2**@DNA21 and **1**@DNA21 assemblies (Eq. S1)[9] was then undertaken.[5d] A 49 % end-to-end ET efficiency was observed for **2**@DNA21 compared with only a 15 % for the **1**@DNA21 (1.0 equiv) assembly. In the case of **1**@DNA21 (3.0 equiv), an end-to-end ET efficiency of 42 % was observed (Table S3) which is less than for **2**@DNA21, thus confirming the superior ET characteristics through the PA-mediated positional control of the YO intercalator.[9]

The application of the PA programming approach was then applied to longer DNA-based photonic wire systems comprising discrete homo- (YO–YO) as well as hetero- (PB–YO and YO–Cy3) ET processes rather than the purely hetero-FRET processes observed for **2**@DNA21. In the case of the DNA55 series (Figure 2b), a two-fold enhancement in Cy3 emission was observed for the DNA assembly comprising four PA binding sites (**2**@DNA55) relative to assemblies comprising an equivalent amount of YO (**1**) (**1**@DNA55, 4.0 equiv). Quantification of the end-to-end ET efficiencies for the DNA55 series showed again the superiority of PA-programming approach with enhanced ET efficiencies for the PA-based **2**@DNA55 assembly (26 %) compared with 12 % and 25 % for **1**@DNA55 (4.0 equiv of **1**) and **1**@DNA55 (12.0 equiv of **1**), respectively (Table S3).

Compared to the equivalent exemplars in the DNA21 series (that is, **2**@DNA21 and **1**@DNA21, 1.0 equiv), **2**@DNA55 and **1**@DNA55 (4.0 equiv) exhibit a different photophysical behavior (Figure 2a compared with Figure 2b). In the DNA55 series, an equivalent amount of YO emission is observed throughout the three assemblies (Figure 2b), whereas a significant decrease in YO emission for **2**@DNA21 relative to the **1**@DNA21 (3.0 equiv) assembly (Figure 2a) is observed. This is indicative of similar levels of energy being trapped at the YO dye step in all three DNA55 examples (Figure 2b) as a consequence of the less efficient YO–YO homo-ET step, relative to the efficient hetero-FRET processes observed in **2**@DNA21. Consistent with the **2**@DNA55 assembly, an enhanced Cy3 emission was also observed for an 80-mer duplex (DNA80) comprising six PA binding sites (**2**@DNA80). In this context a 1.5-fold increase in Cy3 emission was observed relative to the equivalent assembly comprising six equivalents of YO (**1**) (**1**@DNA80, 6.0 equiv; Figure 2c). This equated to an unprecedented end-to-end ET efficiency of 14 % for PA-programmed **2**@DNA80 (6.0 equiv) compared to only 5 % for the YO-PRO based **1**@DNA80 (6.0 equiv) (Table S3).[9]

Time-resolved measurements were finally undertaken in order to investigate the ET rate of the photonic wire assemblies. We measured the fluorescence decay at 570 nm using time-correlated single photon counting (TCSPC) in order to estimate the time required for the excitation energy to arrive at Cy3 after excitation of PB (excitation at 380 nm).[5d] In all three photonic wire assemblies, we observed a consistent trend of PA-programming enhancing the rate of ET typified by shorter average fluorescence lifetimes.[9] For example, significantly shorter lifetimes were observed for **2**@DNA21 (1.5 ns) compared to the control DNA21 (2.7 ns) and **1**@DNA21 (1.0 equiv, 2.4 ns). Consistent with the steady-state data, ET rates of **1**@DNA21 (3.0 equiv, 1.6 ns) approached that of **2**@DNA21 (Figure S6a). This trend was also extended to both the DNA55 and DNA80 series, with **2**@DNA55 (4.0 equiv **2**, 1.8 ns) and **2**@DNA80 (6.0 equiv **2**, 2.9 ns) assemblies exhibiting shorter lifetimes than their equivalent YO (**1**) assemblies **1**@DNA55 (4.0 equiv **1**, 2.9 ns) and **1**@DNA80 (18.0 equiv, 3.1 ns), respectively (Table S4).[9]

In summary, we report the first demonstration of a highly efficient DNA-based photonic wire where energy is transported over an unprecedented distance of 80 base pairs or approximately 27 nm. The key aspect in augmenting ET is defining the location of intercalating YO dyes using DNA-binding PAs. Due to the modularity of the approach, the judicious choice of appropriate fluorophores, and optimization of the interfluorophore distance, increasing the lengths of these photonic wires is indeed possible well beyond our exemplar assemblies highlighted here. Since PAs have the ability to target virtually any DNA sequence, we envisage PA programming becoming a valuable tool in the construction of sophisticated multi-dimensional arrays, motors, and circuits where nanometer-level precision is required.
