# Supplementary material for: Site-Specific Assembly of DNA-Based Photonic Wires by Using Programmable Polyamides
Source: Angew Chem Int Ed Engl. 2011 Feb 25;50(12):2712–5. doi: 10.1002/anie.201006735 (PMC3210824; doi:10.1002/anie.201006735)
Supplement: Supplementary file 1 [file anie0050-2712-SD1.pdf]

Angewandte Chemie
Eine Zeitschrift der Gesellschaft Deutscher Chemiker

Supporting Information
© Wiley-VCH 2011
69451 Weinheim, Germany

Site-Specific Assembly of DNA-Based Photonic Wires by Using Programmable Polyamides**

Wu Su, Markus Schuster, Clive R. Bagshaw, Ulrich Rant, and Glenn A. Burley**

anie_201006735_sm_miscellaneous_information.pdf

Table of Contents

1.0 Abbreviations 1
2.0 Experimental Section 2
2.1 General. 2
2.2 Synthesis of polyamide conjugate (2). 2
2.3 MALDI-ToF Spectrum of polyamide (2) 4
2.4 Reaction scheme of the solid phase synthesis of polyamides 5
2.5 DNA duplex preparation. 6
2.6 UV absorption spectrophotometry. 8
2.7 Fluorescence analysis of DNA binding specificity of polyamide (2). 9
2.8 Steady state Fluorescence Measurements. 11
2.9 Determination of the Quantum Yield of PB-labelled DNA 13
2.10 Calculation of End-to-end energy transfer efficiencies. 13
2.11 Time-Resolved energy transfer measurements. 14
3.0 References 18

1.0 Abbreviations

BTC, Bis-(trichlormethyl)-carbonate; DCM, dichloromethane; DIEA, N-ethyldiisopropylamine; DMF, N, N'-dimethylformamide; DMPA, 3-Dimethylaminopropylamine; Fmoc-D-Dab(Boc)-OH, N-α-(9-fluorenylmethyloxycarbonyl)-N-γ-tbutyloxycarbonyl-D-2,4-diaminobutyric acid; HATU, 2-(1H-7-Azabenzotriazol-1-yl)-1,1,3,3-tetramethyl uronium hexafluorophosphate; NBS, N-bromosuccinimide; TCSPC, time correlated single photon counting; TEA, triethylamine; TFA, Trifluoroacetic acid; THF, Tetrahydrofuran; YO-PRO-1, 4-[(3-methyl-2(3H)-benzoxazolylidene) methyl]-1-[3-(trimethylammonio)-propyl].

2.0 Experimental Section

2.1 General.

All reagents were either HPLC or peptide synthesis grade. DMF, DCM, TFA, THF and DCC were obtained from Acros Organics. BTC, DIEA, and DMPA were purchased from Sigma-Aldrich. 4-Fmoc-hydrazinobenzoyl AM NovaGel resin and HATU were purchased from Novabiochem. Fmoc-D-Dab(Boc)-OH was purchased from ABCR. 1-methyl-1H-imidazole-2-carboxylic acid was purchased from Maybridge Chemicals. 5'- PB labelled DNA strands and YO-PRO-1 (**1**) were purchased from Invitrogen. Non-labelled and 5'- Cy3 labelled DNA strands were purchased from Eurogentec.

Boc-Py-OH and Boc-Im-OH were prepared according to literature procedures.[1]

Analytical and semipreparative RP-HPLC was performed at room temperature on the ULTIMAT 3000 Instrument (DIONEX). UV absorbance was measured using a photodiode array detector at 260 and 310nm. An ACE C18 column (4.6 X 250 mm, 5 µm, 300 Å) was used for analytical RP-HPLC. For semipreparetive HPLC, an ACE C18 column (10 X 250 mm, 5 µm, 300 Å) was used. MALDI-MS was performed on ion trap SL 1100 system (Agilent).

Steady state fluorescence measurements and TCSPC were performed by Horiba Fluorolog 3 fluorimeter.

2.2 Synthesis of polyamide conjugate (2).

Polyamide (PA) was synthesized by solid phase synthesis on the Fmoc-hydrazinobenzoyl AM NovaGel resin (200 mg) at a substitution level of 0.15 mmol/g (Scheme-1).[2] First, the Fmoc group was cleaved with 20% piperidine in DMF. After washing with DMF (4 x 2 mL), a solution of Fmoc-β-Ala-OH (4 eq), HATU (4 eq) and DIEA (8 eq) in DMF was added to the resin. The mixture was shaken for 30 min. After the resin was drained and rinsed with DMF (4 x 2 mL), the resin was capped with 10% pivalic anhydride in DMF for 5min. After washing with DMF (4 x 2

mL), the Fmoc group of β-Ala-OH was cleaved with 20% piperidine in DMF. The resin was then washed with DMF and treated with 1mL dry THF for 2 min. Meanwhile, the following Boc-protected amino acid (4 eq) and BTC (1.3 eq) were dissolved in 1mL dry THF. Collidine (12 eq) was added to the THF solution. After activation for 1min, the suspension was added to the deprotected resin followed by addition of DIEA (8 eq). The mixture was then shaken for 45 min. After the resin was drained and rinsed with DMF (4 x 2 mL), the resin was capped with 10% pivalic anhydride in DMF for 5min. The Boc group was then deprotected using a TFA/Water/Phenol mixture (92.5/2.5/5). This procedure was repeated until a polyamide sequence (Im-Im-Py-Py-γ(Fmoc)-Im-Py-Py-Py-β) bound to the hydrazinobenzoyl resin was obtained. After washing with DCM (4 x 2 mL), the resin was treated with a solution of NBS and pyridine (2.0 eq. each) in 3mL DCM for 10min, drained and washed with DCM (4 x 2 mL). The resin was treated with a solution of 5 equiv. of NH_2-PEG_2-YO (**4**) in DCM for 3 h at room temperature. After the resins were drained and washed with DCM (4 x 2 mL), the filtrates were combined and dried under vacuum. The light-yellow powders were dissolved in 10% MeCN/H_2O/0.1%TFA and purified by semi-preparative reverse phase HPLC (semi-preparative HPLC gradient: started at 10% of B (0.1 TFA in MeCN), hold on 5 min, then increased to 60% of B in 25 min). Lyophilization of the products afforded fine yellow powder (4.2 mg, 9% recovery). PA (**2**) was characterized by MALDI-MS (monoisotopic): calcd m/z 1555.7, found m/z 1555.4 (Figure S1).

2.3 MALDI-ToF Spectrum of polyamide (2)

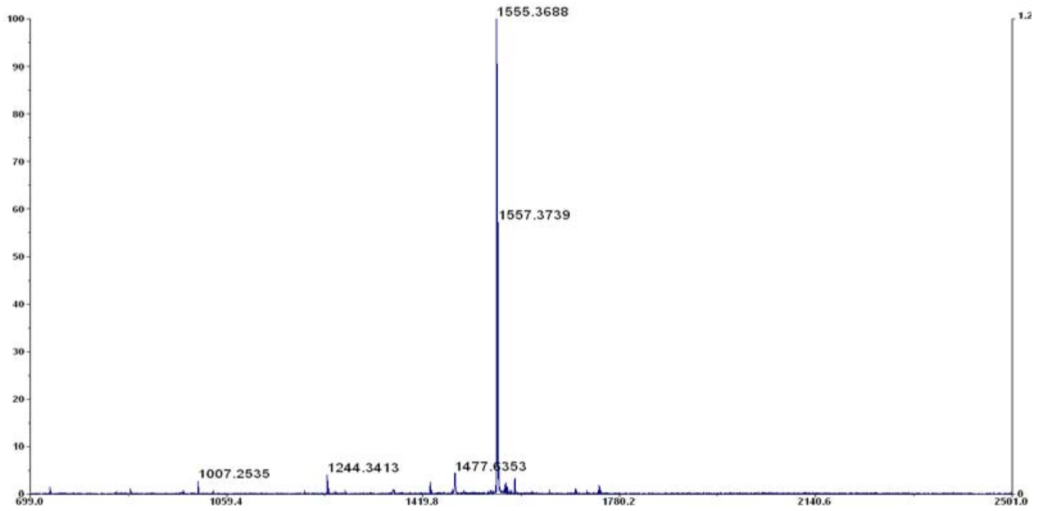

Figure S1. MALDI-ToF mass spectrum of polyamide conjugate (**2**).

2.4 Reaction scheme of the solid phase synthesis of polyamides

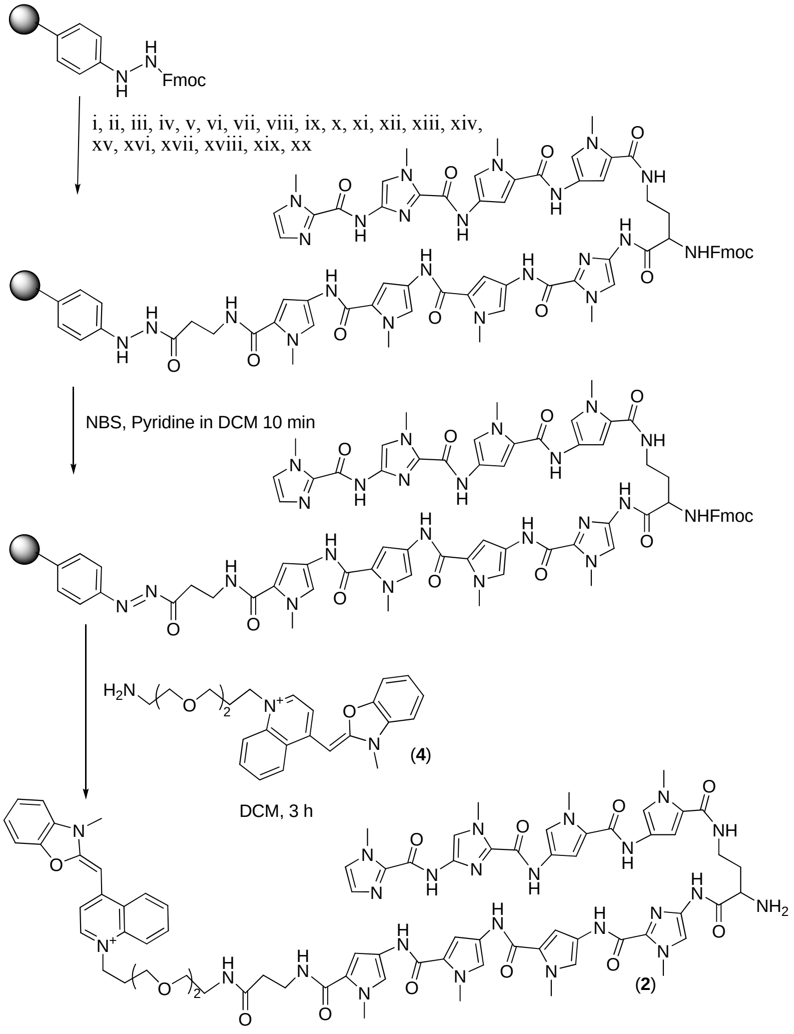

Scheme-1. Synthesis of polyamide conjugate with BTC on hydrazinobenzoyl resin: (i) 20% piperidine/DMF; (ii) Fmoc-β-Ala-OH, HATU, DIEA; (iii) 20% piperidine/DMF; (iv) BocPyOH, BTC, Collidine, DIEA; (v) TFA; (vi) BocPyOH, BTC, Collidine, DIEA; (vii) TFA; (viii) BocPyOH, BTC, Collidine, DIEA; (ix) TFA; (x) BocImOH, BTC, Collidine, DIEA; (xi) TFA; (xii) Fmoc-D-Dab(Boc)-OH, BTC, Collidine, DIEA; (xiii) TFA; (xiv) BocPyOH, BTC, Collidine, DIEA; (xv) TFA; (xvi) BocPyOH, BTC, Collidine, DIEA; (xvii) TFA; (xviii) BocImOH, BTC, Collidine, DIEA; (xix) TFA; (xx) ImOH, BTC, Collidine, DIEA.

2.5 DNA duplex preparation.

Three different lengths of photonic wires were prepared by using fluorescent labelled DNA strands (the polyamide-binding sites are boldfaced):

21-mer duplex (DNA21)

5'- PB – TCG TTA GCA TAT AT***G GAC*** ATA

Complementary strand, 5'- Cy3 – TAT ***GTC C***AT ATA TGC TAA CGA

55-mer duplex (DNA55)

5'- PB – CAT CAT GCA TAT AT***G GAC*** TAT ATA T***GG AC***A TAT ATA T***GG AC***T ATA TAT ***GGA C***AT C

Complementary strand, 5'- Cy3 – GAT ***GTC C***AT ATA TA***G TCC*** ATA TAT AT***G TCC*** ATA TAT A***GT CC***A TAT ATG CAT GAT G

80-mer duplex (DNA80)

5'- PB – CAT CAT GCA TAT ATA T***GG AC***A TAT ATA T***GG AC***T ATA TAT ***GGA C***TA TAT AT***G GAC*** ATA TAT AT***G GAC*** TAT ATA T***GG AC***A TC

Complementary strand, 5'- Cy3 – GAT ***GTC C***AT ATA TA***G TCC*** ATA TAT AT***G TCC*** ATA TAT A***GT CC***A TAT ATA ***GTC C***AT ATA TAT ***GTC C***AT ATA TAT GCA TGA TG

	Sequence
**DNA21**	5’- PB-TCGTTAGCATAT**ATGGACA**TA<br>AGCAATCGTATA**TACCTGT**AT-Cy3-5‘
**DNA55**	5’- PB –<br>CATCATGCATAT**ATGGACT**ATAT**ATGGACA**TATAT**ATGGACT**ATAT**ATGGACA**TC<br><br>GTAGTACGTATA**TACCTGA**TATA**TACCTGT**ATATA**TACCTGA**TATA**TACCTGT**AG-Cy3-5‘
**DNA80**	5’-PB-<br>CATCATGCATATAT**ATGGACA**TATAT**ATGGACT**ATAT**ATGGACT**ATAT**ATGGACA**TATAT**ATGGACT**ATAT**ATGGACA**TC<br><br>GTAGTACGTATATA**TACCTGT**ATATA**TACCTGA**TATA**TACCTGA**TATA**TACCTGT**ATATA**TACCTGA**TATA**TACCTGT**AG-Cy3-5‘

Table S1. DNA sequences used in this study. Boldfaced base pairs correspond to PA binding sites.

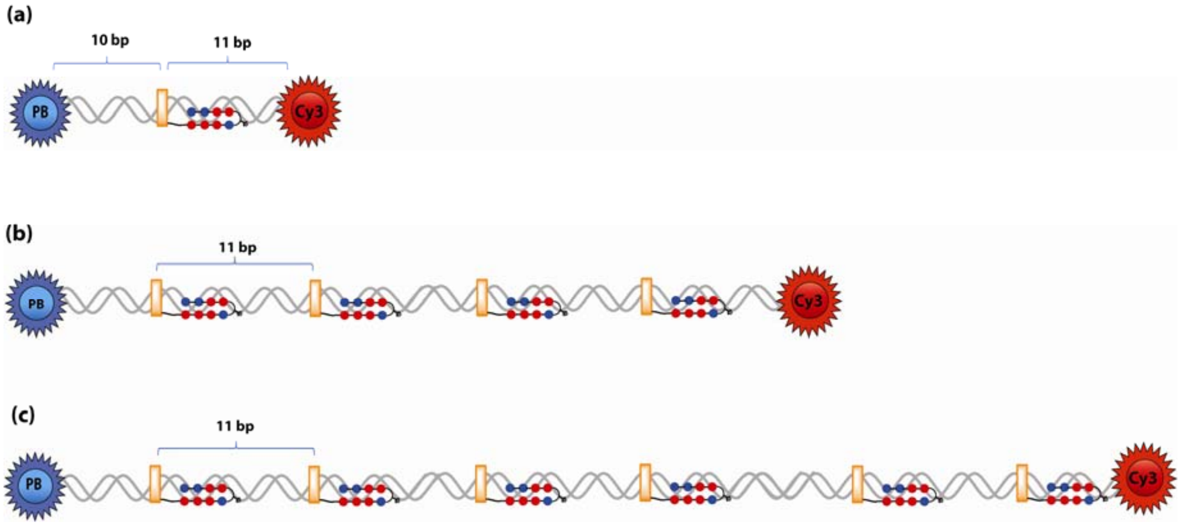

Figure S2. Schematic representation of photonic wire assemblies **(a)** (**2**)@DNA21, **(b)** (**2**)@DNA55, **(c)** (**2**)@DNA80.

Equimolar amounts of PB and Cy3-labelled DNA strands were mixed in sodium phosphate buffer (PBS, 50 mM Na^+, pH7.5) at room temperature to a concentration of 10 μM double strand DNA. Annealing was performed by heating the samples to 85 °C for 10 min, followed by slow cooling to 25 °C over a 6 h period.

2.6 UV absorption spectrophotometry.

Melting temperature analysis was performed on a PerkinElmer Lambda 35 UV/Vis spectrophotometer equipped with a thermo-controlled cell holder possessing a cell path length of 1 cm. A degassed aqueous solution of 10 mM sodium cacodylate, 10 mM KCl, 10 mM $MgCl_2$, and 5 mM $CaCl_2$ at pH 7.0 was used as the analysis buffer.[3] The corresponding DNA duplex and compounds (**1**), (**2**) or (**3**) were mixed with 1:1 stoichiometry to a final concentration of 1 μM for each experiment. Prior to analysis, samples were heated to 90 °C, and cooled to a starting temperature of 25 °C with a heating rate of 5 °C/min for each ramp. Denature profiles were recorded at λ = 260 nm from 40 to 85 °C (Figure S3). The reported melting temperatures were defined as the maximum of the first derivative of the denature profile (Table S2).

**DNA12:** 5’ **CGA TGG ACA AGC** 3’		
	**T_m (˚C)**	**ΔT_m (˚C)**
**-**	55.4	-
**YO-PRO-1 (1)**	55.8	0.4
**PA (2)**	71.4	16.0
**PA (3)**	72.5	17.1

Table S2: Melting temperatures of DNA12 duplex in the absence and presence of (**1**), (**2**) and (**3**).

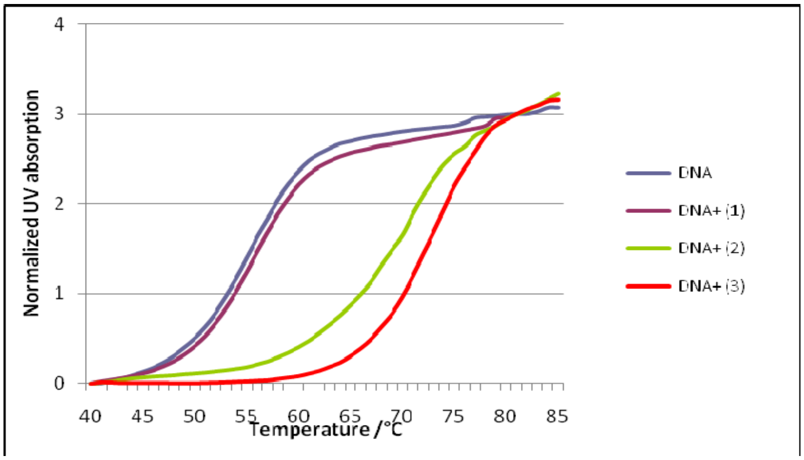

Figure S3: Normalised UV isothermal binding profiles of DNA12 in the absence and presence of (**1**), (**2**) and (**3**).

2.7 Fluorescence analysis of DNA binding specificity of polyamide (2).

Experimental protocol: Two double- strand DNA sequences were prepared in sodium phosphate buffer (PBS, 50 mM Na^+, pH7.5) as a stock solution (10 µM): the match DNA sequences 5' -TCG TTA GCA TAT ATG GAC ATA- 3' and the mismatch DNA sequences 5' -TCG TTA GCA TAT ATG CAC ATA- 3'. In the dark, 500 µL solutions were made to a fixed concentration of 100 nM of (**1**) or (**2**) in PBS as the controls. The experimental solutions were made by 100 nM of (**1**) or (**2**) against 100 nM match or mismatch DNA duplexes. The solutions were gently swirled and then allowed to sit for 5 h before measurements were made. Fluorescence emission profiles were recorded by a Horiba Fluorolog 3 fluorimeter. Emission spectra were collected from 490 nm to 650 nm using an excitation wavelength of 470 nm.

Binding affinity analysis: We investigated the binding and selectivity of (**2**) for the target sequence 5'-ATGGTCA-3' using isothermal binding measurements, which provides a qualitative assessment of the relative binding affinity and sequence selectivity.[4] A high degree of stabilisation (17.1 ˚C) was observed for the control PA (**3**):DNA complex relative to the free DNA12 (1.0 µM) duplex [Table S2] comprising a single PA binding cassette. This significant increase in duplex stabilization was indicative of (**3**) binding to DNA12 with high affinity.[5] A

16.0 ˚C stabilisation was observed for construct (**2**) containing the tethered intercalating YO, suggestive of (**2**) maintaining a high binding affinity for the target sequence within DNA12 despite the tethered YO. In contrast to (**2**) and (**3**), only a slight duplex stabilisation (ΔT_m 0.4 ˚C) was observed when one equivalent of (**1**) was added to DNA12, indicative of the significant influence of the PA element to enhance the thermal stability of the DNA12 duplex (Fig. S4).

DNA selectivity analysis: The selectivity of PAs (**2**) and (**3**) for their target sequence was undertaken using a fluorescence enhancement assay in the presence of DNA21 as well as 21-mer sequences comprising a one base-pair mismatch. YO compounds (**1**) and (**2**) are virtually non-fluorescent in free solution equating to minimal contribution of unbound (**1**) and (**2**) to background fluorescence (Fig. S4).[6] In contrast, an intense emission was measured after the addition of one equivalent of either (**1**) or (**2**) to the matched 21mer DNA duplex sequence, attributed to the intercalation of the YO dye within the DNA duplex.[7]

A 79.3 % drop in YO emission in the presence of the sequence 5' -TCG TTA GCA TAT A**TG** **C****AC** **A**TA- 3' containing a single base pair mismatch (mismatched C underlined) within the main PA core is suggestive of the high sequence selectivity for PA (**2**), and is attributed to the weaker binding affinity of (**2**) for the mismatch site. Fluorescence enhancement was also observed when one equivalent of (**1**) was used in the presence of the 21mer duplex, although no difference in the fluorescence intensity was observed between the matched and mismatched duplexes, thus underpinning the non-sequence selective nature of the YO dye.

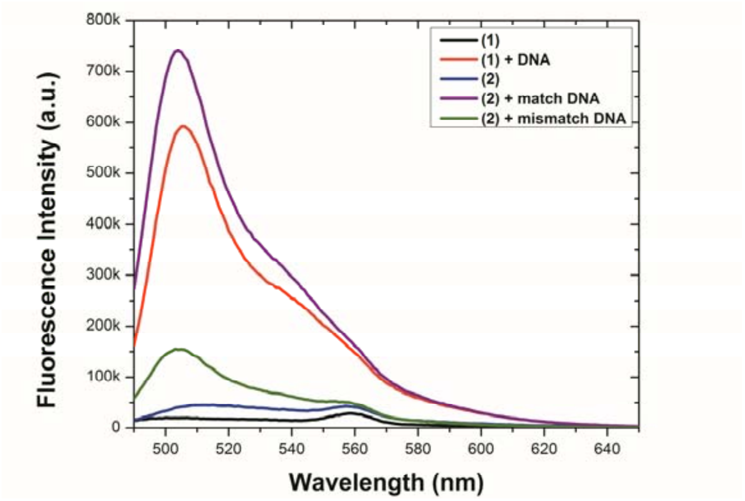

Figure S4. Fluorescence emission profile of (**1**) and (**2**) for the match DNA sequences 5' -TCG TTA GCA TAT ATG GAC ATA- 3' and the mismatch DNA sequences 5' -TCG TTA GCA TAT ATG CAC ATA- 3' [100 nM concentrations. Mismatch base underlined].

2.8 Steady state Fluorescence Measurements.

Steady state fluorescence measurements were performed using a Horiba Fluorolog 3 fluorimeter. In the dark, 500 µL solutions were prepared in PBS buffer (50 mM Na^+, pH7.5), DNA duplex, (**2**) or (**3**) up to a final concentration of 50 nM for the DNA duplex and 50 nM (**2**) or designed concentrations of (**3**) (0, 1, 2, 3, 4 eq of (**3**)). The solutions were gently shaken and then allowed to sit at room temperature for 5 h. The measured sample was placed in a 500 µL quartz cell with 5 mm path length and kept at 20 °C during the measurement. Corrected emission spectra were collected from 400 nm to 650 nm using an excitation wavelength of 380 nm (Figure S5). Repeat scans showed no evidence of significant photobleaching during the experiment. To compare the transfer efficiency of the wires, the number of photons emitted from PB,YO, and Cy3 were deduced from the wire spectra by reconstructing the wire spectra through a linear combination of standard spectra of the individual chromophores W(λ)=A*PB(λ)+B*YO(λ)+C*Cy3(λ). The pre-factors A, B, C were determined from least-square fitting routines and indicate the relative contributions of the individual components to the wire spectra.

(a)

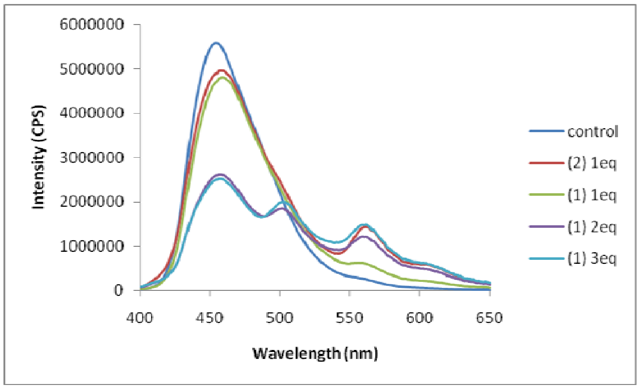

(b)

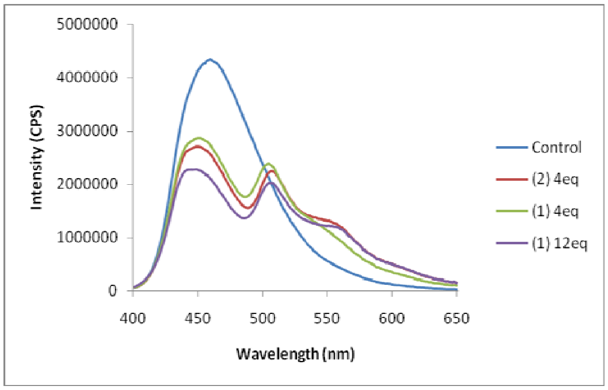

(c)

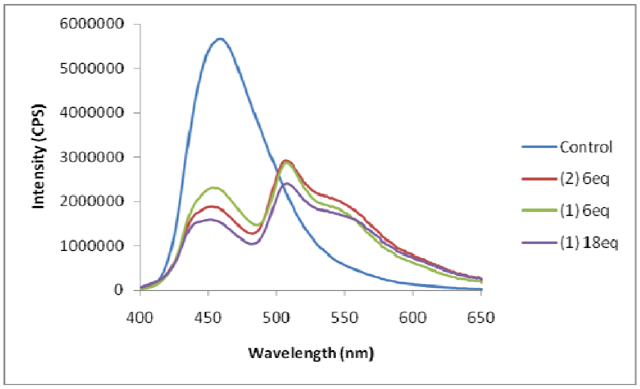

Figure S5: Measured emission spectra of **(a)** DNA21 in the presence of (**2**)(red), 1.0 equiv. (**1**) (yellow), 2.0 equiv. (**1**) (cyan), 3.0 equiv. (**1**) (blue); **(b)** DNA55 in the presence of 4.0 equiv. (**2**) (red), 4.0 equiv. (**1**) (yellow), and 12.0 equiv. (**1**) (cyan); **(c)** DNA80 in the presence of 6.0 equiv. (**2**) (red), 6.0 equiv. (**1**) (yellow), and 18.0 equiv. (**1**) (cyan). Controls are fluorescence-labelled DNA duplexes without intercalators.

2.9 Determination of the Quantum Yield of PB-labelled DNA

The quantum yield of PB-labelled DNA was determined relative to free Pacific Blue dye (Φ_{em} = 0.75)[8] at 20 °C by integration of corrected emission spectra : Solutions for fluorescence measurements typically contained 100nM PB or PB-DNA in PBS buffer (50 mM Na^+, pH7.5). The measured sample was placed in a 500 μL quartz cell with 5 mm path length and kept at 20 °C during the measurement. Corrected emission spectra were collected from 400 nm to 650 nm at an excitation wavelength of 380 nm using a Horiba Fluorolog 3 fluorimeter.

2.10 Calculation of End-to-end energy transfer efficiencies

End-to-end energy transfer efficiencies were calculated according to Hannestad *et al.*[7c] In brief, efficiencies were calculated according to eq. S1, where the number of excited Cy3 molecules was compared to the number of PB molecules upon initial excitation. Dividing the extracted Cy3 emission with the fluorescence quantum yield for DNA-labelled Cy3 (0.16 in double stranded DNA),[9] the total excitation energy transferred from the PB is obtained. The unquenched PB emission is divided by the PB quantum yield (Section 2.9) to afford the number of initially excited PB molecules (eq. S1). The spectra were collected under equivalent conditions with the same chromophore calculations. The model used to calculate energy transfer efficiencies assumes no direct excitation of Cy3 at 380 nm (Table S3). [7c]

$$E = \frac{(F_{CP} - F_C)/Q_C}{F_P/Q_P}$$

Equation S1: F_{CP} represents the integrated Cy3 emission spectra (wavenumber scale) excitation at 380 nm with PB. F_C represents the integrated Cy3 emission spectra (wavenumber scale) excitation at 380 nm without PB. F_p is the integrated Pacific Blue emission from a corresponding wire containing only Pacific Blue. Q_C represents the quantum yield of Cy3-labelled DNA, whereas Q_P represents the quantum yield of PB-labelled DNA (see section 2.9 for quantum yield calculations).

.

DNA construct	End-to-end Energy Transfer Efficiency (%)
**DNA21**	
**(1)**@DNA21 [1.0 equiv. of (**1**)]	15
**(1)**@DNA21 [3.0 equiv. of (**1**)]	42
**(2)**@DNA21 [1.0 equiv. of (**2**)]	49
**DNA55**	
**(1)**@DNA55 [4.0 equiv. of (**1**)]	12
**(1)**@DNA55 [12.0 equiv. of (**1**)]	25
**(2)**@DNA55 [4.0 equiv. of (**2**)]	26
**DNA80**	
**(1)**@DNA80 [6.0 equiv. of (**1**)]	5
**(1)**@DNA80 [18.0 equiv. of (**1**)]	8
**(2)**@DNA80 [4.0 equiv. of (**2**)]	14

Table S3: End-to-end energy transfer efficiency for photonic wire assemblies DNA21, DNA55 and DNA80. Energy transfer efficiencies were calculated according to eq S1.

2.11 Time-Resolved energy transfer measurements.

The fluorescence decays were measured using time correlated single photon counting (TCSPC) on a Horiba Fluorolog 3 TCSPC fluorimeter. A Horiba pulsed diode light source (NanoLED) was used to generate a peak wavelength of 373 nm output pulse. The instrument response function (IRF) was measured using a scattering solution (Ludox provided by Horiba). The TAC range was set at 50 ns. The fluorescence decay of the photonic wire is collected at 570 nm, a wavelength with emission primarily from Cy3 (Figure S5). The measured fluorescence decay is the convolution of the IRF with the intensity decay function. By using Horiba DataStation software, the intensity decay function was fitted using a sum of exponentials, and the goodness of the fit was judged by the reduced X^2 value and visual inspection of the weighted residuals.

The fluorescence decays are fitted using a three-exponential expression, two with positive pre-exponential and one with a negative factor. Excited Pacific Blue or YO act as starting points transferring energy to Cy3, which then decays to the ground state by emission of a photon (Figure S6). The two positive components represent distribution of arrival times from the two chromophores Pacific Blue and YO. The negative exponential is seen in the time-resolved spectra as a risetime, corresponding to the Cy3 fluorescence lifetime[7c] (Table S6).

(a)

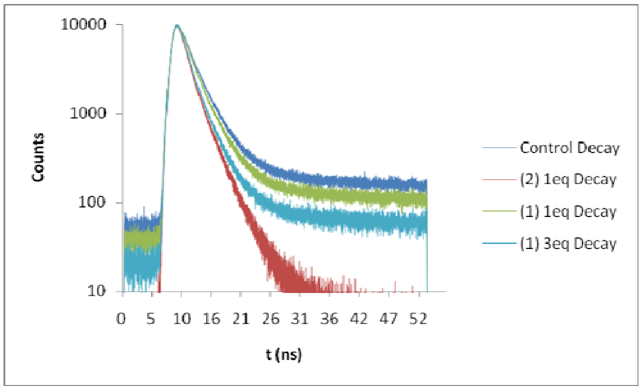

(b)

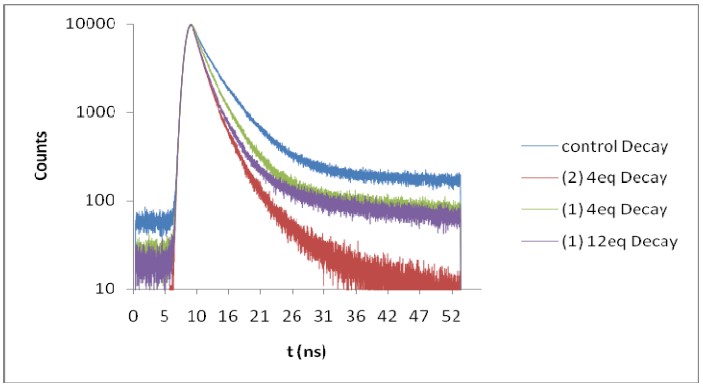

(c)

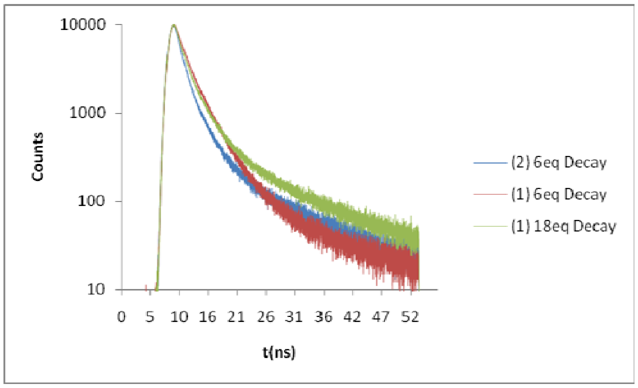

Figure S6: PB fluorescence decay measurements (excitation 372 nm and monitoring emission at 570 nm) of the **(a)** DNA21 [DNA21 control (blue); (**2**)@DNA21 (1.0 equiv., red); (**1**)@DNA21 (1.0 equiv., yellow); (**1**)@DNA21 (3.0 equiv., light blue)]; **(b)** DNA55 [DNA55 control (blue); (**2**)@DNA55 (4.0 equiv., red); (**1**)@DNA55 (4.0 equiv., yellow); (**1**)@DNA55 (12.0 equiv., purple)]; and **(c)** [(**2**)@DNA80 (6.0 equiv., blue); (**1**)@DNA80 (6.0 equiv., red); (**1**)@DNA80 (18.0 equiv., yellow)] assemblies. Controls are fluorescence labelled DNA duplexes without intercalators.

**DNA construct**	**Fluorescence lifetime of photonic wire (ns)**
**DNA21 series**	
DNA21	2.7
**(1)**@DNA21 [1.0 equiv. of (**1**)]	2.4
**(1)**@DNA21 [3.0 equiv. of (**1**)]	1.6
**(2)**@DNA21 [1.0 equiv. of (**2**)]	1.5
**DNA55 series**	
DNA55	3.7
**(1)**@DNA55 [4.0 equiv. of (**1**)]	2.9
**(1)**@DNA55 [12.0 equiv. of (**1**)]	1.8
**(2)**@DNA55 [4.0 equiv. of (**2**)]	1.8
**DNA80 series**	
DNA80	- [a]
**(1)**@DNA80 [6.0 equiv. of (**1**)]	3.2
**(1)**@DNA80 [18.0 equiv. of (**1**)]	3.1
**(2)**@DNA80 [4.0 equiv. of (**2**)]	2.9

Table S4: Fluorescence lifetimes for photonic wire assemblies DNA21, DNA55 and DNA80. [a] No detectable fluorescence emission observed directly from PB to Cy3 using DNA80.

3.0 References

[1] E. E. Baird, P. B. Dervan, *Journal of the American Chemical Society* **1996**, *118*, 6141.
[2] W. Su, S. J. Gray, R. Dondi, G. A. Burley, *Organic Letters* **2009**, *11*, 3910.
[3] C. Dose, M. E. Farkas, D. M. Chenoweth, P. B. Dervan, *Journal of the American Chemical Society* **2008**, *130*, 6859.
[4] D. S. Pilch, N. Poklar, E. E. Baird, P. B. Dervan, K. J. Breslauer, *Biochemistry* **1999**, *38*, 2143.
[5] S. White, J. W. Szewczyk, J. M. Turner, E. E. Baird, P. B. Dervan, *Nature* **1998**, *391*, 468.
[6] C. Carlsson, A. Larsson, M. Jonsson, B. Albinsson, B. Norden, *Journal of Physical Chemistry* **1994**, *98*, 10313.
[7] a)A. Larsson, C. Carlsson, M. Jonsson, B. Albinsson, *Journal of the American Chemical Society* **1994**, *116*, 8459; b)E. J. Fechter, B. Olenyuk, P. B. Dervan, *Journal of the American Chemical Society* **2005**, *127*, 16685; c)J. K. Hannestad, P. Sandin, B. Albinsson, *Journal of the American Chemical Society* **2008**, *130*, 15889.
[8] W. C. Sun, K. R. Gee, R. P. Haugland, *Bioorganic & Medicinal Chemistry Letters* **1998**, *8*, 3107.
[9] M. E. Sanborn, B. K. Connolly, K. Gurunathan, M. Levitus, *Journal of Physical Chemistry B* **2007**, *111*, 11064.
